# A giant lipoma of the parietal peritoneum: Laparoscopic excision with the parietal peritoneum preserving procedure – a case report with literature review

**DOI:** 10.1186/s12893-018-0382-7

**Published:** 2018-08-02

**Authors:** Hanlim Choi, DongHee Ryu, Jae-Woon Choi, Yanjie Xu, Yook Kim

**Affiliations:** 1 Department of Surgery, Chungbuk National University Hospital, 776, 1sunhwan-ro Seowon-gu, Cheongju-si Chungcheongbuk-do, 28644 South Korea; 2 Department of Surgery, Chungbuk National University College of Medicine, Cheongju, South Korea; 3 Department of Radiology, Chungbuk National University Hospital, Cheongju, South Korea

**Keywords:** Giant lipoma, Laparoscopy, Parietal peritoneum, Urinary frequency

## Abstract

**Background:**

Lipomas are very common benign tumors of mature fatty tissue that can occur in any part of the body. However, lipomas of the parietal peritoneum are extremely rare.

**Case presentation:**

A 36-year-old man presented with urinary frequency for 6 months. On computerized tomography of the abdomen and pelvis, a well-defined fatty mass measuring 20 × 11 × 6.5 cm in size, was found in the lower abdominal cavity. We performed a laparoscopic parietal-peritoneum-preserving excision of the mass. The patient was discharged without complications on post-operative day 6.

**Conclusions:**

To our knowledge, a laparoscopic excision with preservation of the parietal peritoneum for a giant parietal peritoneal lipoma has never been reported. Herein, we report a case of a giant lipoma of the parietal peritoneum successfully managed by laparoscopy.

## Background

Lipomas are very common benign tumors of mature fatty tissue that can occur in any part of the body. There are a few reports of giant lipoma of mesentery or omentum [1–4]. However, lipomas of the parietal peritoneum are extremely rare. And there have been no reports of extremely large-sized lipomas of the parietal peritoneum. We describe a case of a giant lipoma of the parietal peritoneum causing urinary frequency secondary to external compression of the bladder. This tumor was successfully managed by laparoscopic excision with preservation of the parietal peritoneum.

## Case presentation

**Fig. 1:**
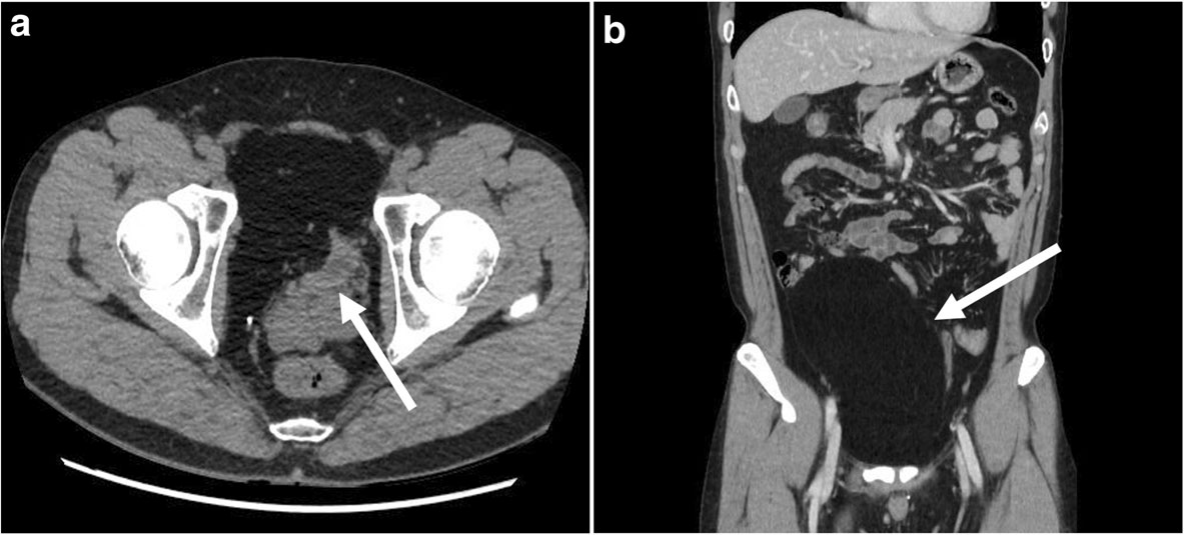
Contrast-enhanced abdomen-pelvis computed tomography scans. **a** Axial view. A mass compressed bladder (arrow). **b** Coronal view. A well-defined homogenous fatty mass measuring 20 × 11 cm in size (arrow)

**Fig. 2:**
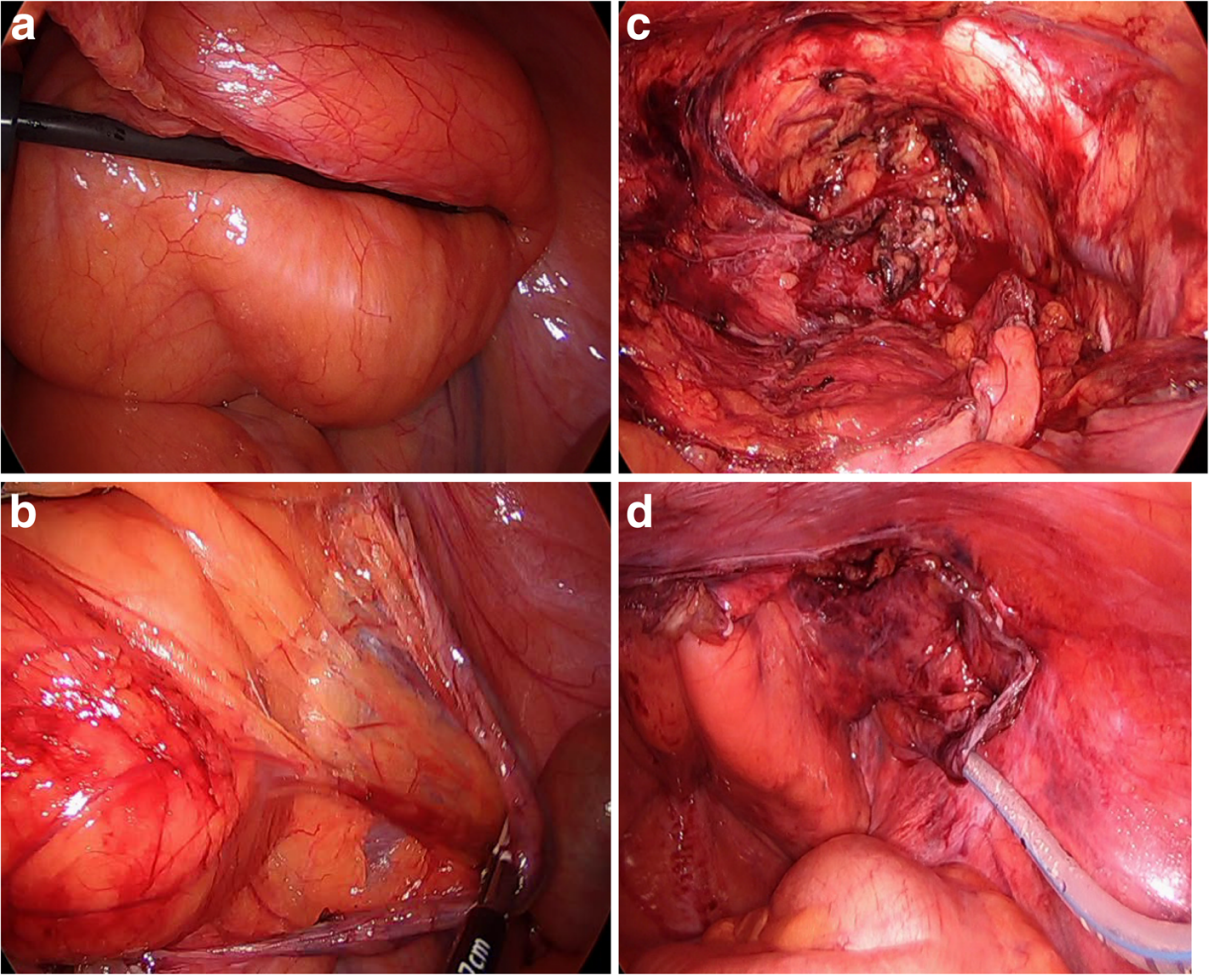
Laparoscopic view. **a** A soft huge mass in the external peritoneal layer was seen in the lower abdomen and was free from other organs. **b** Dissection between mass and peritoneum. **c** Operative field after mass excision. **d** Preserved peritoneum was fixed to abdominal wall using fixation device

A 36-year-old man presented with urinary frequency for 6 months. He had no significant urologic abnormality and no palpable abdominal mass on physical examination. He denied abdominal pain, vomiting, anorexia, or bowel disturbances. There were no specific laboratory abnormalities. The abdomen and pelvis computed tomography scans showed a 20 × 11 cm, well-defined, fatty mass in the abdominal cavity. A mass was located between the abdominal wall muscles and the peritoneum and compressed bladder (Fig.1a, b). We performed surgery, firstly. The reasons are as follows: (1) the mass was just beneath the abdominal wall, (2) the patient had symptom (urinary frequency), and (3) the mass was considered benign from well-demarcate mass with homogenous features on CT scan. We performed a laparoscopic mass excision with preservation of the parietal peritoneum. Two 11-mm ports were inserted, one supra-umbilically, and the other in the left lower abdomen. A 5-mm port was inserted in the right lower abdomen. A huge, freely mobile, soft mass in the external peritoneal layer with no connection to other organs was seen in the lower abdomen (Fig.2a). After demarcating the mass, we excised the parietal peritoneum through the marked line with a monopolar instrument. Next, we dissected the mass from the peritoneum (Fig. 2b). The mass which was excised completely, was placed in a large plastic endopouch-type bag, and extracted through the extended left port site. Finally, the preserved peritoneum was fixed to the abdominal wall using a fixing material with a closed suction drain (Fig. 2c, d). The operative time was 90 min, with no estimated blood loss. The resected specimen size was 22 × 16 × 7.5 cm^3^, and the weight was 942 g. The pathological diagnosis was reported benign lipoma with clear resection margin. The patient was discharged without complications on post-operative day 6.

## Discussion and conclusions

In 2006, the first case of a lipoma of the parietal peritoneum was reported by Barut et al [5]. Since then, only 5 more cases have been reported (Table 1). In previous reports, all the patients presented with abdominal pain. Three cases were presented with right quadrant abdominal pain mimicking appendicitis, and the largest lipoma had a diameter of 6.3 cm [6–10]. Our patient presented with urinary frequency caused by external compression of the bladder. A huge and heavy lipoma measuring 22 × 16 × 7.5 cm3 and 942 g disturbed the filling capacity of the bladder. In 4 previously reported cases, because the lipomas were small in size, they performed a laparoscopic excision of the lipoma and its associated peritoneum [6, 8–10]. In our case, we performed a peritoneal-preserving excision of the lipoma to reduce the pain that we anticipated might be caused by peritoneal resection. Since our patient’s lipoma was large in size, we dissected between the peritoneum and the lipoma, and the peritoneum was preserved with fixation around the abdominal wall. The fixation device (Protack™, Medtronic) which is often used in laparoscopic hernioplasties, was useful for fixation. To our knowledge, a laparoscopic excision with preservation of the parietal peritoneum for a giant parietal peritoneal lipoma has never been reported. This procedure is feasible for decreasing postoperative pain and better cosmetic results.

**Table 1:** Reports regarding the treatment of a lipoma of the parietal peritoneum in the literature

Reference (year)	Age (years)	Sex	Presentation	Surgical procedure	Maximum diameter (cm)
Barut et al. [4] (2006)	67	Female	Abd pain, nausea vomiting	Open	6
Bunker et al. [5] (2013)	34	Female	Abd pain	Laparoscopy	–
Bang et al. [6] (2014)	75	Male	Abd pain, palpable mass	Open	4.5
Shrestha et al. [7] (2014)	32	Male	Abd pain, loss of appetite	Laparoscopy	3
Sathyakrishna et al. [8] (2014)	21	Female	Abd pain	Laparoscopy	–
Salgaonkar et al. [9] (2016)	79	Male	Abd pain	Laparoscopy	6.3
Present case (2018)	36	Male	Urinary frequency	Laparoscopy	22

In conclusion, this case highlights the fact that a giant lipoma of the parietal peritoneum can be an unusual cause of urinary disturbances. Laparoscopic procedures are feasible for the excision of a huge lipomas of the parietal peritoneum, and concomitant preservation of the peritoneum is useful for reducing postoperative pain.

## Abbreviations

A-P CT: Abdominal and pelvic computed tomography
